# Introducing Critical Accounting for Governance as a Tool in Exploring the Commercial Determinants of Health

**DOI:** 10.34172/ijhpm.2023.8128

**Published:** 2023-12-06

**Authors:** Lorna A. Stevenson, Nason Maani, Jonathan R. Olsen

**Affiliations:** ^1^Department of Management, University of St Andrews, St Andrews, UK; ^2^School of Social and Political Science, University of Edinburgh, Edinburgh, UK; ^3^MRC/CSO Social and Public Health Sciences Unit, University of Glasgow, Glasgow, UK

## Introduction and Context

 Commercial determinants of health (CDoH) are defined by the World Health Organization (WHO)^1^ as comprising “private sector activities that affect people’s health positively or negatively.” They “arise in the context of the provision of goods or services for payment and include commercial activities, as well as the environment in which commerce takes place.” Emerging literature examines the various health effects of commercial actors, such as lobbying and policy influence, product marketing and consumption and through system-level effects.^2^ We offer here an additional tool for future research efforts. It has been argued that due to the pervasive nature, scale, and cumulative health effects of commercial actors, there is a need for wider, more interdisciplinary lenses in their study. In their call for more CDoH research the WHO highlighted particular areas of need around “governance considerations, including transparency and accountability,” which we use as our departure point. Taking as a focus the health effects of corporate power, particularly on employees through corporate governance, the key objective here is to outline the relevance of critical accounting as a research tool for CDoH.

 Critical accounting examines corporate power in the contexts of social and environmental justice. Critical accounting scholars recognise that “accounting practices and corporate behaviour are inextricably connected with many allocative, distributive, social, and ecological problems of our era.” Their research “seeks to reformulate corporate, social, and political activity, and the theoretical and practical means by which we apprehend and affect that activity.”^3^ Critical accounting therefore provides approaches and devices which can be used to interrogate health effects of corporations’ power and wider CDoH concerns.

 A recently issued *Business Framework for Health*^4^ report called for businesses to monitor three distinct health impacts of their activities. This Framework highlighted *direct* and *secondary* impacts as well as *external* societal and environmental influences (2021, p. 4). The different aspects of this approach echo Gilmore and colleagues,’^2^ recent model for conceptualising CDoH, which identified “the systems, practices, and pathways through which commercial actors drive health and equity.” They noted the power of corporations in shaping health directly and indirectly, as well as the influence of social norms and the political economy. Our focus on critical accounting builds on their framework by highlighting the scope for political economy and social norms to vary — especially in apparently homogeneous Western high-income countries (HICs) — in ways that affect corporate governance, which may in turn impact employee health through the health effects of power and control. We see critical accounting as a way of initially exploring CDoH effects internal to employing organisations.

 We offer two reasons for our argument. Though the WHO note that “Unsafe or toxic work environments can impact employee mental health”^1^ we argue that even in ostensibly benign work environments, power use may also impact employee mental and physical health.

 Firstly, in the employment context, power links strongly to control, which has been clearly identified as affecting health.^5,6^ We therefore see scope to examine the impact of different power distributions which manifest in control of the employment environment and in turn on employee health.

 Secondly, evidence at national level points to the impacts of political economy^7^ and state governance^8,9^ on population health. We suggest that these analyses are worth extending to the corporate organisation level. We now discuss why corporate governance is a characteristic of corporate power worth considering and then offer a research agenda and data sources informed by critical accounting to begin to examine these aspects.

## Corporate Governance, Forms of Capitalism, and Critical Accounting Insights

 Critical accounting research shows that approaches to corporate governance vary significantly among Western HICs.^10^ Defined as the “set of relationships between a company’s management, its board, its shareholders and other stakeholders,”^11^ approaches to corporate governance are associated with national politics, rather than being simply technical in nature.^12^ The state thus plays an important role in regulating these interactions. HIC state political economies can be characterised as (*a*) Anglo-American or ‘liberal,’ relying on the market mechanism to resolve conflict amongst stakeholders, or (*b*) continental European or ‘co-ordinated’^13^ which traditionally exhibit greater levels of cooperation amongst affected constituent groups, as well as formal and extensive employee influence at macro and micro levels of organisation.^12^

 These distinctions in turn are associated with different approaches to corporate governance. Under the Anglo-American approach, Boards of Directors typically prioritise shareholders’ interests over other stakeholders,’ whereas continental European approaches were customarily designed to balance more sets of stakeholders’ interests. Clear management consequences of these distinct approaches to governing companies were highlighted by an interviewee in a critical accounting study who had experienced each of them. He said “the French system works better in terms of…thinking very carefully about the whole population. In the United Kingdom, you’re very much more driven by shareholder value and so you’re focused very much on driving … profits … and dividends.”^14^ He also relayed an instance of a Swedish Board discussion he was party to which illustrated that there “…people have a ‘very different mindset’”^14^ about appropriate use of corporate profit. We suggest that these differences may in turn affect employee health.

 We now turn to corporate governance and the work environment considering: “there is now a considerable and clear evidence base demonstrating the importance of good quality employment and good health.”^15^ Key to this evidence base is the significance of “a just relation between employers and employees,”^6^ in terms for example, of power and participation. The typical Anglo-American and European corporate governance systems evidence varying power balances and provide different opportunities for employee participation. The Lancet-University of Oslo Commission on Global Governance for Health^16^ identified national health effects arising from ‘dysfunctions of global governance’^16^ including shortfalls in “basic democratic norms, such as equal rights of participation, fair representation, transparency, and accountability.”^16^ At the organisational level, rights to participate in decision-making and governance emerge – or do not–, from the political economy context where the employing organisation is to be found.

 Further, the Organisation for Economic Co-operation and Development’s (OECD’s) Principles of Corporate Governance exhibit an Anglo-American emphasis. These state “The board is not only accountable to the company and its shareholders but also has *a duty* to act in their best interests. In addition, boards are expected to *take due regard* of, and deal fairly with, other stakeholder interests including those of employees, creditors, customers, suppliers and local communities.”^11^ (emphasis added). Although more coordinated economies mandate comparatively greater employee involvement in organisational governance, employees traditionally have very limited information rights; even the International Labour Organization’s (ILO’s) Declaration on Fundamental Principles and Rights at Work fails to include any employee right to participate in decisions around managing, controlling and organising work, or to receive any governance or organisational information. National variations in employees’ power to participate in significant decisions, such as remuneration, organisational restructuring and responses to economic downturns will clearly have different direct impacts on them, and consequent spillover to their families and local communities. For example, Ferguson et al,^17^ showed that in 47 countries Anglo-American corporate governance was associated with both greater income inequality, and greater mortality in children under 5.

 Though the WHO state that “Corporations commonly influence public health through lobbying and party donations” critical accounting scholars Contrafatto et al^18^ also evidenced that companies “worked more successfully in mobilising power strategies ‘behind the scenes’ to bend [EU regulations around governance]…to their (collective) view.” Corporate power has also shaped national policies for governing health. Allen et al,^19^ examined 194 countries’ implementation of WHO’s non-communicable diseases policies and concluded that “Democracy was positively associated with policy implementation, but only in countries with low corporate permeation.” Countries with high corporate influence had poorer WHO non-communicable diseases policy implementation records.

 However even within the European approach there are variations in governance norms. Driven by dominant investor and corporate representatives, there have been recent pressures to change in a more Anglo-American direction, and dilute requirements for worker representation on Boards.^20^ Nonetheless, regardless of their political economy context companies are free to enact more democratic forms of governance than national regulations demand: the co-operative and employee-owned forms are examples. Work in Scotland by McQuaid et al,^21^ on experiences of employee ownership reported workers having: greater control; integral involvement in decision-making; greater awareness around operational and strategic information; and higher levels of health than in the general population. The authors quoted an interviewee: “There is no stress of being in the dark.”

 We therefore suggest that insights and tools of critical accounting be considered to research CDoH, and offer three examples of relevant data and research directions which could be fruitful.

###  Delineating the Possible Permutations and Consequences of Different Forms of Corporate Governance

 Historical, longitudinal, and cross-comparison studies at population level could help us understand the health effects of different corporate governance norms and enable us to begin to unpick these mechanisms. For example, tracing the history of UK corporate governance Sikka^22^ showed that regulation had strengthened corporate power and diluted workers’ rights since the second world war. Examinations of the health effects of this lineage might yield productive insights. Longitudinal and cross-national analyses of population health might draw insight from deploying the OECD Corporate Governance Factbooks and the World Bank national reports on the observance of corporate governance standards and codes,^23^ Eurobarometer statistics on the work environment, and ILO statistics on co-operatives. In addition the Comparative Political Data Set^24^ offers annual “political and institutional country-level data” such as political systems, government composition, macroeconomic data, labour market, industrial disputes and demographics for 36 democratic countries which could be used to explore national governance norms, and population health. European level data on quality of work and employment, industrial democracy, industrial competitiveness, and social justice from 2008^25^ is also available.

###  Widening the Range of Possible Datasets That Could Be Used to Assess CDoH

 Though the association of income inequality with poor population health outcomes is well known, the part played by corporate governance norms does not appear to have been explored in the CDoH literature. In addition to the data sources above, longitudinal, cross-panel and cross-national analyses of population health could include institutional characteristics of trade unions, wage setting, state intervention and social pacts^26^ to assess relative power effects.

###  Understanding Better the Health Intersections Between Corporate Governance and Wider Power Dynamics 

 Data on prevailing beliefs about trust, national norms, experiences of social cohesion and societal balance between collective and individual interests can be found in the World^27^ and European^28^ Values Surveys, and Edelman trust reports.^29^ Triangulation of such data with corporate annual report disclosures, social media engagements, lobbying, political and charitable contributions, board of directors’ biographies could help throw light on companies’ roles in shaping social norms around appropriate uses of corporate power.

 To conclude, the data sources and arguments presented offer intriguing next steps for interested scholars to explore research questions such as ‘What is the evidence for corporate governance as a CDoH on employees?’ with possible consequent public health and corporate governance policy implications. There is a growing appreciation that research and action on the CDoH requires multidisciplinary approaches, including the involvement of new disciplines, methods and data sources. Critical accounting offers a potential tool to the growing armamentarium used in widening and deepening our understandings of corporate power and its impacts on health and prosperity.

## Ethical issues

 Not applicable.

## Competing interests

 Jonathan Olsen is employed by the MRC/CSO Social and Public Health Sciences Unit, University of Glasgow, and supported by the Medical Research Council [Grant number: MC_UU_00022/4] and Chief Scientist Office [Grant number: SPHSU19].
